# Autopsy examination in sudden cardiac death: a current perspective on behalf of the Association for European Cardiovascular Pathology

**DOI:** 10.1007/s00428-020-02949-8

**Published:** 2020-10-28

**Authors:** Jytte Banner, Cristina Basso, Zoe Tolkien, Ivana Kholova, Katarzyna Michaud, Patrick J Gallagher

**Affiliations:** 1 Department of Forensic Medicine, University of Copenhagen, Copenhagen, Denmark; 2 Cardiovascular Pathology Unit, Department of Cardiac, Thoracic and Vascular Sciences and Public Health, University of Padua, Padua, Italy; 3 NutriDART, Bristol, UK; 4 Fimlab Laboratories, Tampere University Hospital, Tampere, Finland; 5 Centre Universitaire Romand de Médecine Légale, Lausanne, Genève Switzerland; 6 Medical Education, Bristol Medical School, Bristol, UK

**Keywords:** Autopsy, Sudden cardiac death, Inherited cardiac conditions—cardiomyopathy

## Abstract

In sudden cardiac death, an autopsy is an essential step in establishing a diagnosis of inherited cardiac disease and identifying families that require cardiac screening. To evaluate aspects of post-mortem practice in Europe, a questionnaire was designed and circulated to both clinical and forensic pathologists. There was a 48% response rate and information was obtained from 17 countries. The results showed a wide variety in the management of sudden cardiac death, with a general tendency towards a lack of thorough investigation. In up to 40% of cases, autopsies were not performed in subjects less than 50 years who may have died from cardiac disease. Reasons for this were lack of finance and lack of interest from police, legal authorities, and doctors. Only 50% of pathologists seem to follow a standard protocol for autopsy examination, apparently due to lack of expertise and/or training. When autopsies were performed, histology and toxicology were almost always taken, genetic studies were generally available and retention of the heart for specialist study was usually permitted. Our results suggest that although the standard of practice is appropriate in many centres, many more cases should have autopsies, especially in sudden deaths in subjects less than 50 years.

## Introduction

Sudden unexpected death is a frequent complication of many forms of cardiac disease. Because there is no internationally agreed definition of sudden cardiac death, the exact incidence is uncertain [1]. Nevertheless, a task force of the European Society of Cardiology has suggested that the incidence ranges from 36 to 128 deaths per 100,000 population per year [2]. Other reviews indicate that the incidence is between 50 and 100 per 100,000 population in a range of different countries [3, 4].

Ischaemic heart disease and heart failure are the most common causes [5, 6], but there are many other cardiac disorders associated with sudden death [7]. Some of these have a defined genetic basis and are thus inheritable [8]. This is especially the case in children and younger adults [9]. Correct autopsy diagnosis is an indispensable step in diagnosing the underlying pathology in sudden cardiac death and is essential for the subsequent screening and investigation of relatives of victims.

Our association (the Association for European Cardiovascular Pathology, AECVP) has previously produced a guideline for the post mortem investigation of patients who die suddenly from cardiac disease [10], and this has been recently updated [11]. The extensive citation and various translations of this document confirms its apparent value for practitioners around the globe. However, such guidelines are of little value if appropriate cases are not selected for post-mortem examination. The clinical, educational and epidemiological value of autopsy examination was addressed by Burton and Underwood in 2007 [12]. They documented a steep decline in non-forensic autopsies in eight different countries, discussed medical and secular reasons for this decline, and emphasized the value of autopsy examination in both undergraduate and post-graduate education. They, and others, have emphasized that autopsies continue to demonstrate important clinically unsuspected pathology [13–15], even in the current era of ‘high-tech medicine’ [16]. A decline in autopsy examinations is of particular concern in patients who may have died suddenly and unexpectedly from potentially inherited cardiac disorders. While these are only a small percentage of all sudden cardiac deaths, the accurate diagnosis in these cases will guide screening of relatives by cardiologists with expertise in inherited cardiac disorders.

In 2010, Michaud and her colleagues published the results of a survey concerning *forensic* practice in cases of sudden cardiac death (SCD) [17]. This demonstrated wide variation in the ordering of autopsies and the standard of investigation. Many of the problems were financial in origin, largely because activities in forensic medicine are often paid by, and dependant on, judicial authorities.

In this report we describe the results of a questionnaire into autopsy practice, sent to forensic and hospital pathologists in different European countries and regions. Particular emphasis was on the management of sudden cardiac death in the young.

## Materials and methods

An on line questionnaire was widely circulated to members of the AEVCP and to National Chairpersons of Clinical and Forensic Pathology Societies. The full text of this questionnaire is available in the supplementary appendix. For the purposes of this study, a young sudden cardiac death victim was a subject less than 50 years of age. Questions addressed the following broad issues:

- Registration of the cause of death
- Overall autopsy rate
- Numbers of sudden cardiac deaths
- Selection of sudden cardiac death cases for autopsy
- Additional investigations after autopsy
- Professional interactions with specialist cardiologists, geneticists and pathologists

The results were analysed, discussed by a writing committee and used for the formulation of recommendations at general assemblies and dedicated meetings of the AECVP.

## Results

A selection of the most important results is presented in Table 1. The following comments are made in summary.

**Table 1:** Summary of questionnaire

Questions	Summary of responses
On which country are your answers based on? (Number of responses per country)	Australia (1); Belgium (2); Czech Republic (1); Denmark (2); Finland (1); France (1); Germany (1); Ireland (1); Italy (4); Netherlands (2); Norway (1); Portugal (1); Russia (5); Slovakia (1); Spain (2); Switzerland (2); United Kingdom (3)
Are the responses based on a whole country, a region of a country or an individual hospital or department	A whole country 10 responses An individual region of a country 15 responses An individual hospital or department 6 responses
What is the general opinion about the registration of causes of death?	Satisfactory, data are always registered 39% Not entirely satisfactory, but data are always registered 55% Not entirely satisfactory, data not always registered 3% Unsatisfactory, data not reliably entered 3%
Are autopsy results considered in official statistics of the causes of death?	Yes 74% No 19% Unsure 7%
What is the overall autopsy rate?	Less than 10% of all deaths 37% Between 10 and 20% of all deaths 26% More than 20% of all deaths 37%
What is the clinical autopsy rate?	< 5% of all deaths 43% 5–10% of all deaths 21% ≥ 20% of all deaths 43%
What percentage of autopsies performed by forensic pathologists are entirely natural deaths?	< 50% of autopsies 23% 50–60% of autopsies 32% ≥ 60% of autopsies 45%
In *forensic* practice who is mainly responsible for ordering an autopsy in a young subject where there are no suspicious circumstances?	Judge 57% Police 37% Medical examiner 3% Doctor attending death 3%
In *clinical* practice who is mainly responsible for ordering an autopsy in a young subject where there are no suspicious circumstances?	Doctor attending death 48% Family doctor 11% Head doctor 7% Judge 19% Others 15%
In *forensic* practice is family consent required in order to perform an autopsy on a young sudden death subject?	No 83% Yes, but can be over ruled by police or judge 14% Yes 3%
In *clinical* practice is family consent required in order to perform an autopsy on a young sudden death subject?	No 43% Yes, but can be over ruled by police or judge 14% Yes 43%
In *forensic* practice who pays for an autopsy in a young sudden death subject?	Judge or legal authority 44% Police 22% Health authority 30% Relatives 4%
In *clinical* practice who pays for an autopsy in a young sudden death subject?	Health authority 80% Judge or other legal authority 8% Relatives 4% Others 8%
If an autopsy is NOT performed in a young subject where cardiac disease is thought to be the cause of death what is (are) the reason(s). *Multiple responses included*	No finance for forensic autopsy 9 responses No finance for clinical autopsy 5 responses No interest from police or judge 16 responses No interest from clinical team 8 responses No interest from clinical pathologist 7 responses Other reasons 7 responses
In a young sudden cardiac death autopsy do your pathologists follow AECVP guidelines?	In every, or almost every, case 30% In more than half of cases 20% In less than half of cases 27% Never, or almost never 23%
When pathologists do not follow guidelines what is the reason(s) *Multiple responses included*	Pathologist has insufficient knowledge or training 12 responses Pathologist has insufficient time 7 responses Insufficient funds 7 responses No police or legal interest 3 responses Other reasons 6 responses
Do clinical and/or forensic pathologists have established collaborations with specialists in cardiovascular pathology?	Yes, through recognized centres 29% Yes, on a personal basis 49% Yes, but practice varies 19% No 3%
Are pathologists permitted to retain the full heart for further examination or referral	Yes,67% Yes, sometimes 23% Never or hardly ever 10%
Do pathologists require family consent to retain the heart?	Yes, 17% No, 80% Sometimes, 3%
Do pathologists order toxicology	Always, or in more than 50% of cases 68% Never, or in less than 50% of cases 19% Only when indicated by post mortem findings 13%
Do pathologists perform histology on the heart of a young sudden death subject	In every, or almost every, case 81% In more than 50% of cases 13% Less than 50% of cases 6%
Do pathologists make a histological examination of the conduction system	In all, or more than 50%, of cases 24% In less than 50% of cases 41% Never 35%
Do pathologists make immunohistochemical studies of cardiac tissue	In all, or more than 50% of cases 10% Never, or in less than 50% of cases 37% Only when appropriate on the basis of histology 53%
Do pathologists store frozen tissue for future genetic analyses?	In every case 30% In more than 50% of cases 27% In less than 50% of cases 20% Never, or almost never 23%
Having retained frozen samples are your pathologists, or their clinical colleagues able to perform genetic analyses?	Yes for a large panel of relevant genes, 68% Yes, for a limited panel of genes 16% No, 16%
Who is responsible for ensuring that the relatives of a young sudden cardiac death subject are referred to a cardiologist or clinical geneticist?	Pathologists who performed the autopsy 32% Family doctor of the deceased 42% Judge or police 6% Other responses 20%
Do pathologists have contact with the families of young sudden cardiac death subjects?	In almost every case, 19% in > 50% of cases, 13% In < 50% of cases, 29% Never or almost never, 39%
Do pathologists have contact with the family doctor of young sudden cardiac death subjects?	In > 50% of cases 17% In < 50% of cases 33% Never, or almost never 50%
Do pathologists have established collaborations with cardiologists and/or geneticists with expertise in inhertited cardiac conditions?	Yes with cardiologists and cardiologists 49% Yes but with cardiologists only 3% Yes, but practice variable 19% No established collaboration in the majority of departments 29%
How often do pathologists receive clinical or molecular information from cardiologists or geneticists about relatives of sudden death subjects?	Often 30% Sometimes 23% Hardly ever 30% Never 17%
How often do pathologists receive critical comments from cardiologists or clinical geneticists about the standard of post mortem reports?	Often 13% Sometimes 3% Hardly ever 37% Never 47%

• In all but two questions only single answers could be given and the results are expressed as percentages. For 2 questions multiple answers were permitted and results are expressed numerically

AECVP Association for European Cardiovascular Pathology

### Respondents

Thirty-one completed questionnaires were returned (48% response rate). Responses were obtained from 17 different countries. The population on which a response was based ranged from 0.5 to 82 million. The majority of respondents (21/31) were forensic pathologists. Twenty respondents worked in a university setting.

### Numbers of autopsies performed

The number of autopsies performed varied considerably. In Denmark, Germany, Italy, Norway, Spain and the Netherlands, ≤ 5% of all deaths were autopsied in a region of Switzerland, in Slovakia, Ireland and the UK between 10 and 20%, in Finland 23% and in separate regions of Russia between 30 and 60%.

In most countries or regions, forensic autopsies were performed in much less than 5% of all deaths. The rate was between 5 and 10% in Finland, Portugal, Spain, in a region of the Czech Republic. In contrast forensic autopsies were performed in more than 30% of deaths in three separate regions of Russia. Many autopsies performed by forensic pathologists were considered to be natural deaths.

Clinical autopsies, performed by hospital-based pathologists, were infrequent, usually much less than 5% of all deaths. Exceptions included Finland (7%), Ireland (20%) and specific regions of Switzerland (20%), Belgium (23%) and a region of Russia (64%).

### Autopsies in sudden cardiac death

Only nine respondents were able to provide detailed information on the numbers of sudden cardiac deaths in subjects of all ages. Denmark, Finland and the St. Petersburg region of Russia have broadly similar populations of ~5 million but reported 3500, 7500 and 12,000 sudden cardiac deaths per year. Results from individual regions in the Czech Republic, Russia, Spain, and the UK, with populations between 0.8 and 1.65 million, gave an autopsy confirmed sudden death rate of ~50 per 100,000 per year.

In subjects less than 50 years of age half of the respondents indicated that between 10 and 12.5% of all deaths were sudden cardiac deaths. National responses from Denmark, Finland, Germany, and the Netherlands suggested that sudden cardiac death rates in subjects less than 50 years were 12.7, 16.0, 5.1 and 13.5 per 100,000 population per year. When asked the question: A subject < 50 years dies suddenly. Cardiac disease is thought to be the cause of death. How often is an autopsy performed? responses were as follows: always, or almost always, 35%; more than 50% of cases, 27%; less than 50% of cases, 35%; never, 3%.

### Autopsy guidelines and reports

The guidelines for autopsy practice in sudden cardiac death produced by our Association were always followed by 30% of respondents and a further 20% did so in “more than 50%” of cases. The remainder did so in “less than 50%” of cases or never followed guidelines. Reasons for not following guidelines included a lack of knowledge or training of pathologists (12 responses) and insufficient time and/or funds (7 responses each).

### Histology, toxicology and molecular studies

Standard histology and toxicology were performed in most, but not every, autopsy. DNA was extracted from post mortem tissues in the majority of cases and could be screened for a wide number (68%) or a limited range (16%) of genetic abnormalities (see Table for details).

## Discussion and recommendations

### Autopsies in adult subjects

The objective of the present study was to obtain information about the conduct of sudden cardiac death autopsies. Most of the information that we obtained was from countries in the European Union, but we also obtained information from members of our Association practising in other parts of Europe and elsewhere (see Table 1).

Our study has demonstrated that an autopsy is often ***not*** performed after the sudden death of a previously fit adult of less than 50 years of age. Lack of finance and lack of interest (from police, legal authorities and doctors), or a combination of these issues were some of the most important reasons for this. In our survey 30% of respondents always followed autopsy guidelines and a further 20% in half of cases. Low resources and insufficient knowledge or expertise were the most common causes of non-compliance. These reasons are not unique to autopsy practice. For example, in a large recent European analysis of challenges in implementing guidelines to prevent spread of multidrug resistant gram negative infections [18] the number of infection control staff, lack of dedicated educational programmes and the number of clinical staff were identified as important reasons for non-compliance.

On a more positive note histology and toxicology were performed in the majority of autopsies, genetic analysis of retained tissue was widely available and the retention of the heart was usually possible. There was also ready access to the opinion of specialists in cardiovascular pathology. However, post-mortem tissues were not always retained frozen for future genetic studies, and this is a matter of particular concern. We also suggest that pathologists should be in more frequent contact with the relatives and family doctors of the deceased to explain the results of the autopsy examination and the need for referral to a specialist cardiologist.

### Forensic autopsies

Our results indicated that European forensic pathologists perform large numbers of autopsies on patients who die of natural causes, emphasizing the increasing role they are now playing in autopsy pathology in Europe. Their practice may have indications extending beyond the identification of the cause of death and may require imaging techniques and more advanced sampling for toxicology, biochemistry and microbiology. Cardiovascular disease can have an important role in many accidental deaths including road traffic collisions [19], trauma [20] or in drowning or water immersion [21].As in clinical autopsies in forensic cases where the cause of cardiac arrest is uncertain a full histological examination with toxicology and retention of tissues for future genetic studies is warranted [11, 22, 23].

### Autopsies in children

An Australian study of subjects 1–35 years of age confirmed that SCD occurs in very young subjects [9]. Of the 490 cases, 50 were aged between 1 and 5 years. Paediatric pathologists are likely to perform these autopsies and when non-cardiac causes have been excluded should follow AECVP guidelines for dissection of the heart. Retention of tissue for genetic studies is especially important in children as they are more likely to have potentially inherited cardiomyopathies or structurally normal hearts that may have underlying genetic abnormalities [9, 22]. Cases of the Sudden Infant Death Syndrome (SIDS) are usually dissected by specialist pathologists, following protocols that aim to identify risk factors for asphyxia and potential infective agents [24, 25]. In a recent multi-centre study of 419 cases of SIDS 12.6% had a “potentially informative” variant in a targeted analysis of 90 genetic heart disease susceptibility genes [26]. However only 4.3% had an “immediately actionable” abnormality.

### Limitations, key concerns and recommendations

We were disappointed that only 48% of questionnaires were returned and accept that this is a potential limitation of our study. Furthermore three or more responses were received from Russia, Italy, the Netherlands and the UK. The majority of respondents were forensic pathologists, probably a reflection of the role they play in the delivery of autopsy services. In addition, we suspect that those who did complete the questionnaire may have had a particular interest in autopsy pathology and cardiac disease.

Despite these reservations, it is of particular concern that our study demonstrated important deficiencies in current autopsy practice. These included a low autopsy rate in some centres, a lack of training and expertise in cardiovascular pathology and a lack of knowledge, or failure of application, of anatomical and molecular guidelines for sudden cardiac death autopsies. A larger number of responses from pathologists working in district hospitals would have been desirable, but we doubt that this information would have demonstrated an improvement in overall practice.

Our association recommends that a full autopsy should be performed in any subject less than 35 years who dies suddenly and unexpectedly. Where there is any suspicion of cardiac disease, an anatomical and molecular autopsy should be performed [11, 22, 23]. We believe that it is desirable that this policy is extended to subjects less than 50 years of age. Age limits are necessarily arbitrary, but it is the common experience of specialists in cardiovascular pathology that some cases of sudden cardiac death due to inherited cardiac disease present in patients between 35 and 50 years [27, 28]. In contrast only occasional cases of sudden cardiac death due to inherited causes present in those > 50 years [27]. A recommendation that all sudden unexpected deaths should have autopsies is unrealistic. However, if there is a family history of inherited cardiac disease and/or premature sudden death, an autopsy should be considered in patients of any age.
